# Secondary metabolite induced tolerance to *Fusarium oxysporum* f.sp. *cubense* TR4 in banana cv. Grand Naine through *in vitro* bio-immunization: a prospective research translation from induction to field tolerance

**DOI:** 10.3389/fmicb.2023.1233469

**Published:** 2023-11-28

**Authors:** T Damodaran, Maneesh Mishra, M Muthukumar, Shailendra Rajan, Kavita Yadav, Alok Kumar, Prasenjit Debnath, Sangeeta Kumari, Popy Bora, Ram Gopal, Sandeep Kumar

**Affiliations:** ^1^ICAR-Central Institute for Subtropical Horticulture, Lucknow, Uttar Pradesh, India; ^2^ICAR-Central Soil Salinity Research Institute, Lucknow, Uttar Pradesh, India; ^3^Assam Agricultural University, Jorhat, Assam, India; ^4^Krishi Vigyan Kendra, Masodha, Acharya Narendra Deva University of Agriculture and Technology, Kumarganj, Ayodhya, Uttar Pradesh, India

**Keywords:** *Fusarium oxysporum* f.sp. *cubense* TR4, banana, wilt disease, tissue culture, bioimmune, LC-MS, tolerance mechanism, field trails

## Abstract

An innovative tissue culture mediated incorporation of metabolite-based biomolecule (Bio-immune) at *in vitro* stage itself in banana cv. Grand Naine was developed and validated for the production of *Fusarium oxysporum* f.sp. *cubense* TR4 tolerant plantlets. The novel bio-immune formulation developed by us, exhibited a significant antifungal potency against *Foc* TR4 with a high percent inhibition (100%) at a 2.5% concentration of bio-immune on the 5^th^, 7^th^, and 9^th^ DAI. Bio-immune integrated during *in vitro* shoot proliferation stage in banana cv. Grand Naine recorded significant enhancement in the growth of roots and shoots. Bio-immune (0.5%) fortified media produced 12.67 shoots per clump whereas control registered only 9.67 shoots per clump. Similarly, maximum root numbers (7.67) were observed in bio-immune plants which were significantly higher over control (5.0). The bio-immunized banana transplants recorded a higher survival rate (97.57%) during acclimatization as compared to the control (94.53%). Furthermore, evaluation of the bio-immunized plants in pot experiments revealed that unimmunized plants treated with *Foc*TR4 (TF) exhibited mortality between 60 and 90 days. On the 90th day after planting, a high mean disease severity index (DSI) of 3.45 was observed with unimmunized plantlets while the bio-immunized plants (TFBI) and ICAR-FUSICONT treated plants (TFTR) showed substantially reduced DSI (0.20 and 1.00) compared to *Foc*TR4 treated control (TF). Significant increases in polyphenol oxidase (PPO), peroxidase (POD), β-1,3-glucanase, phenylalanine ammonia-lyase (PAL), chitinase activities, and enhanced phenol contents were recorded in bio-immunized plants compared to unimmunized plants. Field experiments at two different locations in Bihar, India revealed that bunch weight, no. of hands/bunch, and no. of fingers/hand of bio-immune treated plants were significantly higher compared to the control.

## 1 Introduction

*Musa* spp. bananas are a common economic crop grown in tropical and subtropical areas (Wang et al., 2021), and one of the most popular exported fruits that serve as a staple diet for >400 million individuals globally (Sandborn, 2022). The banana crop economy has endured significant losses owing to an outbreak of *Fusarium* wilt since 1860, caused by *Fusarium oxysporum* f.sp. *cubense* race 1 (*Foc* R1). The replacement of *Gros Michel* by Cavendish cultivars, resistant to *Foc* R1, solved the problem of Fusarium wilt on banana to a great extent (Buddenhagen, 1990; Ploetz, 2015). Intensive cultivation of Cavendish cultivars in the sub-tropics of South Africa, Australia under low temperatures (10 to 35°C) resulted in a *Foc* variant as Race4 showing extensive wilting (Su et al., 1986; Bubici et al., 2019). *Fusarium* wilt induced by *Foc* TR4 is the most damaging banana disease, restricting production worldwide (Ploetz and Pegg, 2000; Damodaran et al., 2020). Of late, there has been a severe epidemic of *Foc* TR4 in the northern part of India across provinces of Bihar and Uttar Pradesh, reducing banana production in one of the biggest banana-producing belts of the country (Damodaran, 2018). The herbaceous perennial nature of banana and the polycyclic behavior of disease contribute for huge epidemics there by causing dramatic economic losses with period of time (Ismaila et al., 2023). The colonization tendency of *Foc*TR4 was proliferating more promptly and with increased pathogenicity.

Many initiatives have been performed to mitigate the impact of *Fusarium* wilt worldwide. Several scientists throughout the globe have explored a variety of strategies for the management of *Foc*TR4 including chemical and biological methods, altering cultural practices, and developing tolerant or resistant lines through breeding. The capacity of *Foc* TR4 to persist without its host is a crucial issue impeding the effective treatment of this disease. Chemical treatment methods including soil soaking with fungicide and corm injection failed to offer long-term disease control, while their recurrent usage has also posed environmental issues (Getha and Vikineswary, 2002). Soil fumigants, flood fallowing, and agricultural sanitation are used to moderately manage a vast region (Ploetz et al., 1990). Feasibility of biological suppression of *Foc* TR4 pathogen using beneficial microbes such as *Trichoderma reesei* (Damodaran et al., 2020) and *Streptomyces* sp. (Cao et al., 2022) are considered as a viable option to check the pathogen spread for a specific period of time. Strengthening the biological control measures for long-term sustainability in the management of the disease needs supplemental research approaches that can induce tolerance in the host (Kavino et al., 2008).

Bio-priming is a process where the fixed concentration of an effective microbe with proven growth promotion and disease control characteristics are primed (treated) to the rooted tissue culture plantlets that come out of the laboratory for primary and secondary hardening in the nursery stage to develop tolerant and disease-free material (Reddy, 2012; Jebakumar and Selvarajan, 2018). It is carried out at the late primary hardening stage in the poly house and continues in the secondary hardening phase before the plant goes for planting. Bio-priming of tissue culture plantlets to induce tolerance during primary and secondary hardening stages using bioagents was attempted earlier (Kavino et al., 2007a; Deshmukh et al., 2020). However, the response at the field level over the period of time was variable (Damodaran et al., 2021).

With the growing need for developing eco-friendly alternatives to disease management, in the recent past, the identification of antifungal chemicals and metabolites from the rhizosphere and endophytic biome of plants have been used for inducing adaptive innate immune responses in plant systems against specific pathogens (Fadiji and Babalola, 2020). Chemical metabolites- based induction of immune responses against specific pathogens and developing acquired or induced disease resistance in plants is a new alternative area of research in terms of environmental safety. Bio-immunization is a process where the bio-active antifungal metabolites of microbes with antagonistic or growth-promoting properties are obtained through selective extraction and integrated in the Murashige & Skoog (MS) rooting media in the laboratory to the shoot tips that are to be initiated for rooting at a known concentration to aid in inducing the plant innate immune response to biotic stress. The present study provides a unique one such bio-immuno formulation obtained from *Foc* TR4 antagonist *Trichoderma reesei* (CSR-T-3) attempted to study the efficacy in the development of immuno-booster antifungal compounds in the plants while they are in the shooting and rooting phase under aseptic laboratory conditions without contaminating the media and not creating somaclonal variation between the plantlets grown in MS shooting and rooting media. This could be a plausible novel approach for combating the frightening scenario brought on by the unexpected breakout of *Foc* TR4. In this context, a novel technique for bio-immunization of the multiple shoots generated during the *in-vitro* mass multiplication of banana plants cv. Grand Naine was attempted through a patent-protected protocol in the current study to impart field tolerance to the toxins generated during the pathogenesis of the host by the pathogen.

## 2 Materials and methods

### 2.1 Collections of *Fusarium oxysporum* f.sp. *cubense* TR4 and *Trichoderma reesei*

The biocontrol agent *Trichoderma reesei* was obtained from the Soil Microbiology Laboratory, ICAR-Central Soil Salinity Research Institute, Regional Research Station, Lucknow, India. It was previously isolated from the rhizospheric soil of banana cultivar G9 grown in salt-affected soil of Uttar Pradesh, India (Damodaran et al., 2020) and reported to control *Fusarium* wilt disease of Bananas (Damodaran et al., 2020; Yadav et al., 2021). Similar to this, the virulent *Fusarium oxysporum* f.sp. *cubense* tropical race 4 strain was obtained from the Soil Microbiology Laboratory, ICAR-CSSRI, RRS, Lucknow, India, which had made the disease’s first report of an epidemic in India (Damodaran et al., 2018). Pure cultures of isolates were maintained for further studies.

### 2.2 Development of bio-engineered biomolecule

A biomolecule formulation from *Trichoderma reesei* “BIO-IMMUNE” was developed and patented (Patent File No. 202111003761) and the process of *in vitro* bio-immunization for immunizing the banana tissue culture plantlets during *in vitro* organogenesis was also patented. Both the biomolecule and the technique have been jointly patented by ICAR-CSSRI and ICAR-CISH, Lucknow, India. The culture was inoculated in potato dextrose broth (PDB) and incubated at 35°C for 72 h and the cell free extract (CFE) was collected. CFE was precipitated with alcohol, purified, characterized using LC-MS analysis (as described in Section 2.8), and fractionated for isolating the biomolecule. A series of experiments were conducted to standardize the percentage of liquid formulation to be integrated into the MS rooting medium for *in vitro*-bioimmunization. Around 0.5% of the formulation was integrated into the MS rooting medium based on the factor that the plantlets did not produce any somaclonal variations.

### 2.3 Antagonistic potential of bio-immune formulation on *Fusarium oxysporum* f.sp. *cubense* TR4

The antifungal efficiency of bio-immune formulation against *Foc*TR4 was estimated using the poisoned food technique. Bio-immune formulation was added to PDA medium at 0, 0.5, 1, 1.5, 2.0, and 2.5% concentrations individually. The plates were inoculated with *Foc*TR4. The plate with 0% concentration was considered as control. All the plates were incubated at 28 ± 2°C for 9 days, and the growth was observed on the 5th, 7th, and 9th day after inoculation (DAI).

The estimated percent growth inhibition was calculated according to the following equation:


$$\%\;\text{Inhibition}=\frac{\left(A-B\right)}{A}X\;100$$

where,

A is the radial growth of *Foc* TR4 in control plates, and.

B is the radial growth of *Foc* TR4 in treated plates.

Three replicates of this *in vitro* antagonistic potential experiment were performed and the data were statistically evaluated using SPSS software utilizing analysis of variance (ANOVA) and Tukey HSD.

### 2.4 Effect of bio-immunization on *in vitro* rhizogenesis, shoot proliferation, and hardening of G-9 banana variety

The explant of G9 cultivars were collected from banana plants grown in the *Foc*tolerant region of Bihar. New suckers were treated with bavistin for about 30 min before being cut off at a length of around 3–5 cm and thoroughly cleaned for 10 to 15 min under running water. All traces of bavistin were removed completely by repeated washing beneath a flowing tap water 4–5 times, then with distilled water. Shoot tips were prepared by trimming the corm and outer leaf sheath from the suckers. These shoot tips were exposed to the HgCl_2_0.1% solution for five minutes and washed with sterile distilled water 4–5 times under aseptic conditions. All explants were grown on MS media (Murashige and Skoog, 1962) and bio-immunized with a bioengineered tukey HSD (*p* ≤ 0.05) biomolecule. The unimmunized plants were considered as control. The pH was adjusted to 5.7 before autoclaving. All culture bottles were incubated at 25 ± 2°C with cool white fluorescent tubes for a 16 h photoperiod of light and dark. The materials were sub-cultured in the same medium at regular intervals of 25 days in order to produce several shoots. For measuring the growth parameters, the plantlets were carefully taken out of the culture flasks, and the roots were washed gently under running tap water to remove any agar that had become attached to the roots. The growth parameters root length, no. of roots and shoots, no. of leaves, average leaf length, and the weight of roots and shoots when they are fresh and dry were measured. The samples were dried at 65°C for 96 h in a hot air oven to determine the dry weight of root and shoot.

For banana hardening, coco peat and soil were mixed in a 1:1 ratio and filled in plastic pro-trays for primary, then polythene bags of size 7*4 for the secondary hardening phase. The details of treatments used were T1 = bio-immunized plants and T2 = unimmunized (control) plants. The experiment was replicated six times having a complete randomized design.

Tap water was filled into glass jars containing tissue-cultured plantlets that were being grown in a rooting media of the respective treatments. Plantlets were carefully removed from culture bottles and placed inside a plastic tub filled with water to avoid wilting and excessive transpiration. Plant roots were meticulously freed from the nutritional medium. Banana explant was transferred in plastic pro-trays for primary hardening and was sprinkled with water every hour on the first day after transplanting and then every three hours for the next week. Plants were then marked with tags, and the necessary observations were taken. After 30–35 days single banana explants were transferred in each plastic bag of corresponding treatments for secondary hardening. For the initial hardening trials, the Gothic arch-style greenhouse was built using a UV-stabilized poly-sheet, a thermal shade net, thermometers, hygrometers, and lux meters to measure temperature, humidity, and light intensity. They need to undergo many physiological and anatomical modifications to establish themselves under greenhouse conditions. The platelets are progressively exposed to conditions of decreasing humidity (70%) and rising light intensity after being exposed to the maximum humidity (90%) and diffused light, respectively. To accomplish this, they were transferred to shade houses for secondary hardening.

For secondary hardening, a green shade net with a 50% light cutoff and micro-sprinklers were used for secondary hardening trials.

### 2.5 *In vivo* assay *Fusarium* wilt’s suppression under pot conditions

Using a completely randomized design (CRD) with three replicates, a systematic pot experiment was conducted in June–July 2021 at the ICAR-CSSRI, RRS, Lucknow, India. For the pot experiment, clayey loam soil (pH 7.45, E.C. 0.32 dS/m, and organic carbon 3.0 g/kg) was taken from the Institute’s research farm, sieved through a 20 mm sieve, and then sterilized in an autoclave at 121°C for 30 min for three consecutive days. The 5-day-old fungal culture was inoculated in potato dextrose broth (PDB), and then incubated for an additional 5 days at 28°C in an incubated shaker to create a fungal pathogen culture. The broth was filtered through two layers of cheesecloth to produce spore suspension. Using a hemocytometer, the filtrate spore count was changed from 10^5^ to 10^6^. About 100 mL of the diluted filtrate was poured into sterile soil. For the experiment, uniform-height secondary hardened banana plantlets of the G9 variety (susceptible and bio-immunized) were chosen. The plants were immersed for 30 min in pathogen spore suspension containing 10^6^ conidia/ml before planting, as described by Pérez-Vicente et al. (2014). Four treatments *viz.*, T1 = bio-immunized plants with *Foc* TR4 (TFBI), T2 = unimmunized plants with *Foc* TR4 treated with ICAR-FUSICONT (TFTR), T3 = unimmunized plants with *Foc* TR4 (TF), and T4 = unimmunized plants without *Foc* TR4 (TC). The experiment was designed in a complete randomized form. At 0 and 90 days after planting, the phenological indicators banana plantlets, such as plant height (cm), plant girth (cm), and no. of leaves were observed. The disease scale-based scoring was done as described for *Foc* TR4 scoring by Damodaran et al. (2020). Analysis of variance (ANOVA) was used to statistically examine the data, and Duncan’s multiple range tests (*p* < 0.05) were used to compare means.

### 2.6 Biochemical analysis of defense-related enzymes and phenol content

One gram of third leaf samples was homogenized in 2 mL of 0.1 M sodium citrate buffer (pH 5.0) and centrifuged for 20 min at 4°C at 10,000 rpm. The supernatant containing crude enzyme extract was used for testing the activity of chitinase (Boller and Mauch, 1988), and β- 1, 3-glucanase (Pan et al., 1991).

For the assessment of peroxidase (PO) method from Hammerschmidt et al. (1982), phenylalanine ammonia lyase (PAL) method from Ross and Sederoff (1992), and polyphenol oxidase (PPO) protocol by Mayer et al. (1965) were adopted. The enzymes were extracted in 0.1 M sodium phosphate buffer (pH 7.0). Using Zieslin and Ben-Zaken (1993) methodology, the total phenol content was estimated and expressed in terms of catechol equivalents g^−1^ of protein. The assays were carried out for the T1, T2, T3 and T4 treatments.

### 2.7 SEM analysis of bio-immunized and unimmunized control plants of banana

Scanning electron microscopy of the bio-immunized and unimmunized banana plants’ root sections was done as per the protocol described by Kumar et al. (2018). Sections of the sucker area adjoining the roots were fixed in 3% glutaraldehyde dissolved in 0.1 M phosphate buffer (PH 7.0). The specimens were dehydrated using acetone at a series of concentrations (30 to 90% in increments of 10%) for 20 min, dried for 30 min, and mounted on a steel stub with double-sided carbon tape. The samples were finally coated with a film of gold–palladium alloy under vacuum and observed with a scanning electron microscope (SEM, Fei Quanta 200) at Babasaheb Bhimrao Ambedkar University, Lucknow, India.

### 2.8 Liquid chromatography coupled with mass spectrometric analysis of the leaf samples of bio-immunized and unimmunized control banana plants

Four different treatments *viz.*, T1 = bio-immunized plants with *Foc* TR4 (TFBI), T2 = unimmunized plants with *Foc* TR4 treated with ICAR-FUSICONT (TFTR), T3 = unimmunized plants with *Foc*TR4 (TF), and T4 = unimmunized plants without *Foc* TR4 (TC) plant samples underwent LC-MS analysis, to find key chemical components causing host tolerance in the pot trial. The freshly harvested leaves were chopped into little pieces, properly cleaned under running water, rinsed with sterile distilled water, and then dried in the shade for 15 to 20 days at room temperature. For LC-MS analysis 100 g of dry weight plant material was individually extracted with ethanol and ethyl acetate solvents (400 mL) overnight at room temperature. The Whatmann No. 1 filter paper was used to filter all the LC-MS extracts and was concentrated using a rotary evaporator. A small portion of the prepared plant extracts were collected in Eppendorf and sent to CSIR-Central Drug Research Institute, Lucknow, India for further LC-MS analysis.

The ESI-LC-MS of the TLC purified samples were analyzed using a Micromass Quattro II triple quadrupole mass spectrometer with a JASCO PU-980 HPLC Pump at CSIR-Central Drug Research Institute, Sophisticated Analytical Instrumentation Facility (SAIF), Lucknow, Uttar Pradesh, India for analysis. The water absorption (250_4.6mm_5I) column was used with acetonitrile: water +0.1% formic acid solvent system, Gradient elution was performed at 1.0 mL/min. The photodiode array was monitored at 200–650 nm and recorded at 220 nm. The mass spectra were scanned in the range 80–1,000 DA in 2.5 S. The ESO capillary was set at 3.5 kv and the cone voltage at 40 V. The m/z spectral chromatographs were analyzed, key metabolites were predicted based on already published reports, m/z database and presented for comparative analysis of the status of secondary metabolites in the banana leaf samples collected from bio-immunized and unimmunized grand nine plants both grown in similar sodic soil conditions. Key metabolites were identified based on previously published data and tabulated for comparison analysis between samples of all four treatments using the m/z spectral chromatographs.

### 2.9 Field study for evaluation of bio-immunized plants on disease-affected hotspots of Bihar

Field research was done as Inter-institutional research project of ICAR at two locations Nirpur, Purnia district (25^0^ 43′ 38.626 N, 87^0^ 08′ 18.455 E) and Dighari, Katihar district (Bihar, India) (25^0^ 35′ 38.872 N, 87^0^ 29′ 55.824 E) during 2020–21, 2021–22 to evaluate the field-level efficiency of bio-immunized banana plantlets in controlling *Fusarium* wilt. The selection of study area was based on the severity of the disease. Previously the technological intervention using ICAR-FUSICONT in one of the two locations i.e. Dighari of Katihar district was made through the formation of community groups of the cultivators from the affected areas. In the tissue culture facility of the ICAR-CSSRI, RRS, Lucknow, Uttar Pradesh, bio-immunized disease-free banana plantlets of cv. Grand Naine (G-9) were grown, and 50 plants per replication were utilized for each treatment. The four treatments were, T1- (TBI) treatment includes bio-immunized G-9 banana plantlets planted in a *Foc* infested field, T2- (TTR) includes un-immunized banana G-9 plantlets grown in *Foc* infected field and treated with the ICAR-FUSICONT (*Trichoderma reesei* (CSR-T-3)), T3- (TBITR) includes bio-immunized G-9 banana plantlets grown in a *Foc* infected field treated with the ICAR-FUSICONT, and T4- (TC) included un-immunized G-9 banana plantlets grown in a *Foc* infected field. After five and nine months of planting, the plants under the treatment T2 and T3 were inoculated with 500 mL/plant with 3% ICAR-FUSICONT formulation. Using IBM SPSS statistical software, the recorded growth and yield data were subjected to an ANOVA test (*p* < 0.05) for each parameter. The disease incidence was calculated based on the symptoms that appeared on diseased plants. The disease incidence was calculated by: $DI=\frac{n}{N}X\;100.$

where, DI – Disease incidence; *n* = no. of plants diseased; *N* = total no. of plants observed.

## 3 Results

### 3.1 *In vitro* bioassay of bio-immune formulation against *Foc*TR4

**Figure 1:**
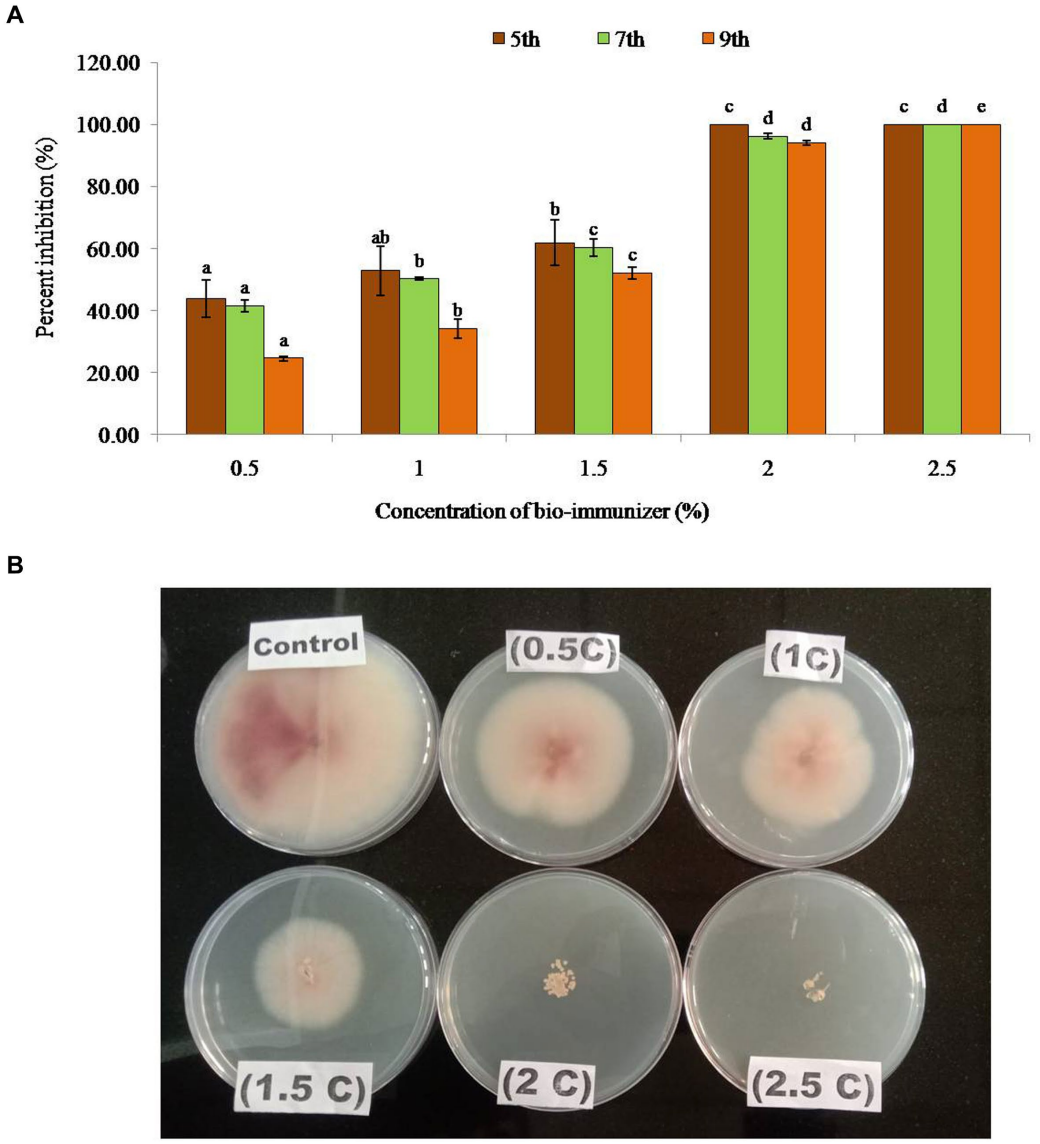
**(A)** Percent inhibition of bio-immunizer on the radial growth of *Fusarium oxysporum* f.sp. *cubense* TR4 under *in vitro* conditions. Values with standard deviation (mean ± SD) are displayed for *n* = 3. Bar graph with different lowercase letters is used to indicate significantly different values by Tukey HSD (*p* ≤ 0.05). **(B)** Effect of bio-immunizer of the radial growth of *Fusarium oxysporum* f.sp. *cubense* TR4 under *in vitro* condition. This study evaluated the antifungal efficiency of a bio-immune formulation against *Fusarium oxysporum* f.sp. *cubense* TR4, utilizing the poisoned food technique. Different concentrations of the bio-immune formulation (0 to 2.5%) were individually incorporated into Potato Dextrose Agar (PDA) medium, and were subsequently point inoculated with *Foc* TR4, with the 0% as the control. The plates were incubated for 9 days at 28 ± 2°C, and the fungal growth was monitored on the 5th, 7th, and 9th day after inoculation (DAI). Results indicated a significant antifungal potency (*p* ≤ 0.05) of the bio-immune formulation against *Foc* TR4 at various concentrations compared to the control. The observed inhibition zone diameter ranged from 2.70 cm to 6.03 cm. On the 9th DAI, the highest radial growth (6.03 cm) was observed at a concentration of 0.5%, while the minimum radial growth (0.47 cm) was recorded at the 2.0% concentration. Remarkably, no radial growth of *Foc* TR4 was observed at the 2.5% concentration of the bio-immune formulation. Moreover, the data exhibited a concentration dependent relationship, whereby an increase in the bio-immune formulation concentration resulted in a significant (*p* ≤ 0.05) augmentation in the percent inhibition of *Foc* TR4. Notably, the highest percent inhibition (100%) was observed at the 2.5% concentration of the bio-immune formulation, followed by the 2.0% concentration, respectively. These findings showed that the bio-immune formulation possesses strong antifungal activity against *Foc* TR4, with potential implications for the management of Fusarium wilt disease in bananas.

Bio-immune formulation exhibited a significant antifungal potency (*p* ≤ 0.05) against the *Foc* TR4 at different concentrations, compared with the control. The inhibition zone diameter ranged between 2.70 cm – 6.03 cm in different concentrations. On the 9th day after inoculation, the maximum radial growth (6.03 cm) was observed at a concentration of 0.5%, while minimum radial growth (0.47 cm) was recorded at 2.0% concentration (Table 1). Whereas no radial growth was observed in *Foc* TR4 at a 2.5% concentration of the bio-immune formulation, the data showed that with the increase in the concentration of the bio-immune formulation the percent of inhibition of *Foc*TR4significantly (*p* ≤ 0.05) increases. The highest percent inhibition (100%) was observed at 2.5% concentration of bio-immune on the 5th, 7th, and 9th DAI followed by 100% on the 5th DAI, 96% on the 7th DAI, and 94% on the 9th DAI at 2.0% concentration, respectively, (Figure 1). It was observed that minimum concentration (0.5%) significantly (*p* ≤ 0.05) reduced the growth of *Foc* TR4 by 44% (5th DAI), 42% (7th DAI), and 25% (9th DAI) respectively.

**Table 1:** Impact of *in-vitro* bio-immunization on rhizogenesis and shoot proliferation under laboratory.

Variables	Rhizogenesis		
	Bio-immunization	Control	*F value*
Root length (cm)	4.63 (±0.51)	3.73 (±0.21)	7.924
Number of leaves	4.37 (±0.42)	3.70 (±0.61)	2.689
Number of roots	7.67 (±1.21)	5.00 (±0.87)	9.595
Root fresh wt. (mg)	59.67 (±4.04)	39.60 (±6.22)	21.959
Root dry wt. (mg)	12.67 (±1.53)	6.67 (±1.06)	31.167

Variables	Shoot proliferation		
	Bio-immunization	Control	*F value*
Number of shoots	12.67 (±0.86)	9.67 (±1.70)	7.404
Avg. leaf length (cm)	3.43 (±0.40)	5.43 (±0.67)	19.780
Shoot fresh wt. (mg)	2.03 (±0.57)	3.70 (±0.36)	18.382
Shoot dry wt. (mg)	0.13 (±0.17)	0.33 (±0.03)	80.00

Each treatment was the mean of three replicates (bottles) per treatment with five roots/shoots per replicate. Values indicate the mean ± SD. There is significant difference between the treatment at *p* < 0.05.

### 3.2 Effect of bio-immunization on *in-vitro* rhizogenesis, shoot proliferation, and hardening stages of tissue culture banana plants of G-9 banana variety

The result, which is represented in Table 1, makes it clearly evident that the biomolecule was highly effective in promoting the *in vitro roots* and shoot growth of banana plantlets. The highest significant (*p* ≤ 0.05) values for root length (4.63 cm), number of leaves (4.37), roots (7.67), fresh root weight 59.67 mg, and dried root weight traits (12.67 mg), were observed in bio-engineered tissue culture banana plantlets compared to control for the same traits (3.73 cm, 3.70, 5.00, 39.60 mg, and 6.67 mg, respectively).

Similar to this, the results indicated that mean shoot proliferation rates of bio-engineered biomolecule were significantly (*p* ≤ 0.05) higher after the basal cycle. The bio-engineered banana tissue culture plantlets showed significantly (*p* ≤ 0.05) higher values (12.67, 3.43 cm, 2.03 mg, and 0.13 mg) for no. of shoots, average leaf length, fresh root weight, and shoot dry weight, respectively, compared to control 9.67, 5.43 cm, 3.70 mg, and 0.30 mg.

Before transplanting the banana tissue culture for the hardening process, the soil’s pH and Ec were observed to be 7.6 and 0.13 ds/m, respectively. During the primary hardening, some growth metrics were assessed for banana transplants (Table 2). Overall, all observed vegetative growth metrics included, plant height (12.53 cm), root length (10.07 cm), plant girth (2.47 cm), no. of primary roots (6.47), no. of leaves (6.27), and leaf area (125.40 cm^2^) were significantly (*p* ≤ 0.05) higher in bio-immunized banana transplants compared to control by 28.17, 6.65, 21.86, 14.99, 14.35, and 6.56%, respectively. The survival rate (%) for the initially acclimated plantlets is the most crucial variable since it shows how well the bio-engineered biomolecule assists the primary acclimation of the banana transplants. Banana transplants that have been acclimated had a greater survival rate of up to 97.57% for the bio-immunized banana transplants compared to the control (94.53%) respectively.

**Table 2:** Impact of *in-vitro* bio-immunization on growth of primary and secondary hardening banana tissue culture plantlets.

Variables	Primary hardening		
	Bio-immunization	Control	*F value*
Survival (%)	97.57 (±0.49)	94.53 (±1.17)	17.180
Plant height (cm)	12.53 (±3.02)	9.00 (±0.44)	4.030
Root length (cm)	10.07 (±0.31)	9.40 (±0.26)	8.163
Plant girth (cm)	2.47 (±0.21)	1.93 (±0.12)	15.059
Number of primary roots	6.47 (±0.38)	5.50 (±0.35)	10.646
Number of leaves	6.27 (±0.15)	5.37 (±0.40)	13.018
Leaf area (cm^2^)	125.40 (±5.06)	117.17 (±3.79)	5.081

Variables	Secondary hardening		
	Bio-immunization	Control	*F value*
Survival (%)	93.23 (±1.07)	89.97 (±0.12)	27.560
Plant height (cm)	19.33 (±5.16)	15.53 (±1.27)	1.534
Root length (cm)	22.10 (±1.39)	16.00 (±0.60)	48.747
Plant girth (cm)	4.67 (±0.25)	3.60 (±0.20)	33.032
Number of primary roots	10.57 (±1.31)	9.80 (±0.10)	1.029
Number of leaves	6.40 (±0.17)	6.13 (±0.12)	4.923
Leaf area (cm^2^)	265 0.93 (±2.63)	163.83 (±3.62)	1562.602

Values are the means of three replicates with sample size *n* = 5. Values indicates the mean ± SD. There is significant difference among the treatments at *p* < 0.05.

Similarly, during secondary hardening, bio-engineered biomolecule significantly (*p* ≤ 0.05) enhanced all the vegetative growth parameters (Table 3) *viz.* plant height (19.66%), root length (27.60%), plant girth (22.91%), no. of primary roots (7.28%), no. of leaves (4.22%), and leaf area (38.39%), respectively, compared to control. Likewise, the survival rate in unimmunized (control) plants (89.97%) was low compared to bio-immunized (93.23%) plants by 3.50%, respectively.

**Table 3:** Effect of bio-immunization and ICAR-FUSICONT [*Trichoderma reesei* (CSR-T-3)] treatment on *Fusarium* wilt management and growth of banana plantlets under field experiment in *Foc* TR4 infected fields.

Treat.	Plant height (cm)	Plant girth (cm)	No. of leaves/Plant	3rd leaf Length (cm)	3rd leaf breath (cm)	Bunch wt. (kg)	No. of hands/bunch	No. of fingers/hand
Location 1: Nirpur, Purnia district, Bihar, India								
TBI	249.30c (±6.76)	68.70c (±3.18)	10.00b (±0.79)	222.40c (±5.71)	72.00c (±1.34)	28.25b (±1.59)	11.25b (±0.85)	17.50c (±1.05)
TTR	238.90b (±3.01)	57.95b (±5.17)	8.10a (±0.72)	200.40b (±6.12)	65.53b (±3.60)	28.70b (±1.13)	9.25a (±0.72)	15.20b (±0.83)
TBITR	257.30d (±5.41)	70.80d (±5.00)	11.15c (±0.88)	233.45d (±2.93)	73.65d (±1.35)	30.85c (±1.60)	12.30c (±0.66)	17.45c (±1.10)
TC	222.65a (±5.25)	53.65a (±1.50)	7.65a (±0.59)	186.65a (±4.76)	59.20a (±2.40)	16.19a (±1.11)	9.10a (±0.72)	14.60a (±1.10)
Location 2: Dighari, Katihar district, Bihar, India								
TBI	230.05b (±9.27)	66.45c (±3.84)	10.55b (±1.10)	196.10b (±5.21)	82.60d (±4.26)	25.65b (±2.13)	11.30b (±1.17)	18.95d (±3.14)
TTR	238.75c (±12.27)	62.60b (±2.16)	10.45b (±2.70)	207.75c (±11.76)	73.55b (±7.76)	27.10c (±2.05)	11.10b (±1.21)	15.00b (±1.30)
TBITR	249.40d (±4.12)	63.80b (±2.48)	12.35c (±1.04)	228.35d (±5.24)	80.05c (±5.80)	31.70d (±2.47)	12.40c (±0.75)	17.15c (±1.50)
TC	187.55a (±6.35)	55.15a (±2.30)	7.65a (±0.59)	147.45a (±6.26)	53.45a (±1.23)	14.13a (±2.47)	9.10a (±0.72)	12.90a (±0.79)

TFBI, Treatment Fusarium and bio-immunized; TTR, Treatment Trichoderma; TBITR, Treatment bio-immunized and Trichoderma; TC, Treatment control (No inoculations). Values are the means of ten replicates with the sample size *n* = 5. Means in the columns followed by the distinct letters are significantly different according to Duncan’s multiple range test at *p* = 0.05. The standard deviation of the mean is indicated by values in parentheses.

### 3.3 *In vivo* evaluation of bio-immunized plants under pot conditions against *fusarium* wilt

**Figure 2:**
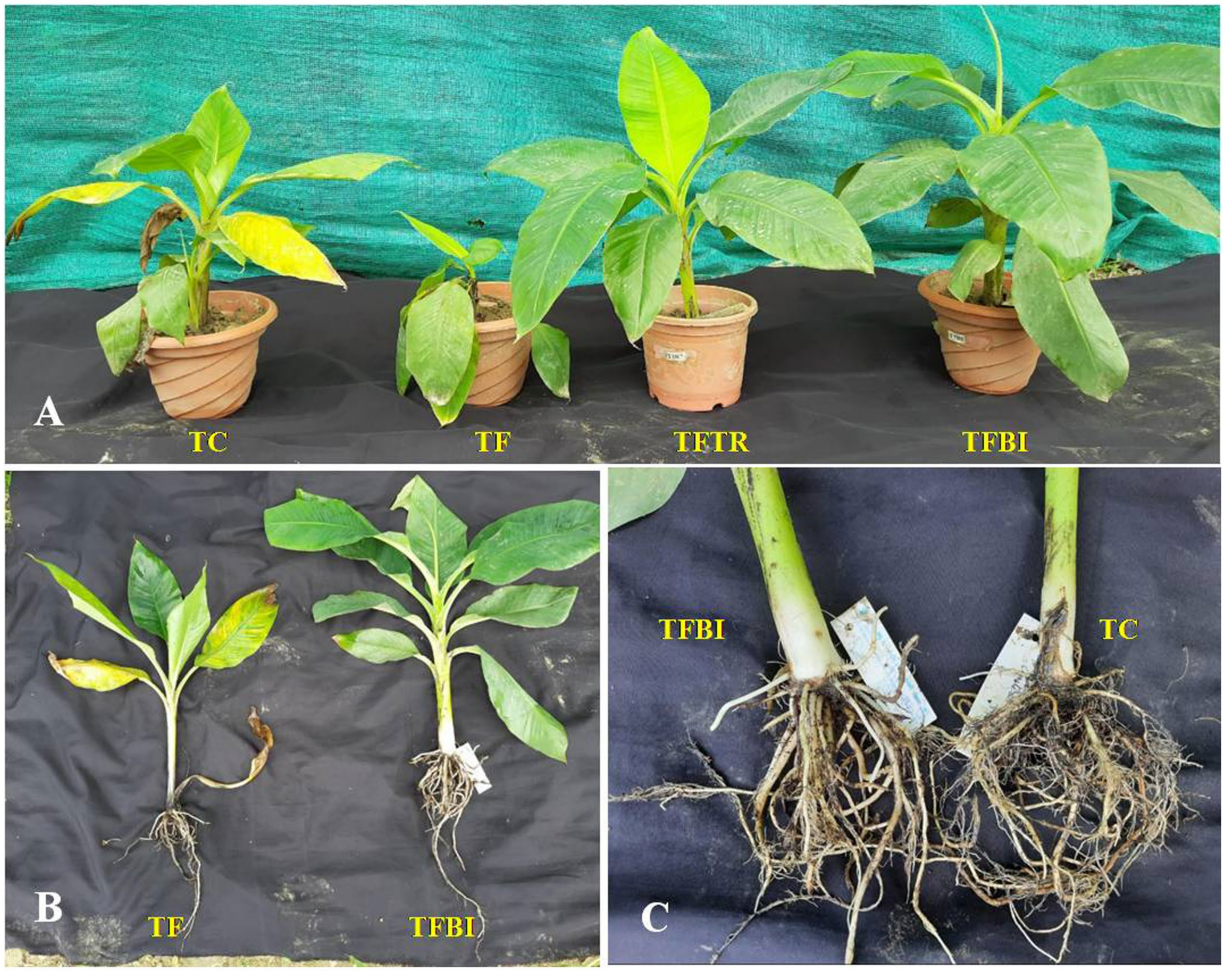
Superiority of *in vitro* bioimmunized plants over other treatments in terms of plant growth and root characters. **(A)** Plant growth enhancement of tissue culture raised banana plantlets under various treatments. TFBI, treatment *Fusarium* and bio-immunized; TFTR, treatment *Fusarium* and *Trichoderma*; TF, treatment *Fusarium* alone; TC, control; **(B)** complete TC plants; TF: Effect of *Foc* TR4 is visible in the plant growth showing leaf yellowing and poor root growth. TFBI- healthy plant with higher visible biomass along with sound root system; **(C)** magnified view of root system: TFBI – with dense and multiple adventitious roots; TC – shallow root system with lower no of adventitious roots.

The use of bio-immunized plants considerably accelerates banana plant development, as seen by an increase in height and girth of the banana plants as compared to the control, in addition to suppressing *Fusarium* wilt (Table 2; Figure 2).

The ultimate height and girth immunized plants (TFBI) were measured to be 38.54 cm and 5.80 cm, respectively, were higher than those of the ICAR-FUSICONT-treated plants (TFTR) (34.78 cm and 5.22 cm), *Foc*TR4 treated control (TF) plants (31.34 cm and 3.74 cm), and untreated control (TC) plants (26.90 cm and 3.06 cm) respectively. Remarkably ICAR-FUSICONT-treated plants showed a significant (*p* ≤ 0.05) difference in percent increment in plant height and girth (TFTR) (20.43 and 28.46%) followed by bio-immunized plants (TFBI) (18.92 and 28.39%). The higher no. of leaves (7.00) was recorded in bio-immunized plants (TFBI) followed by untreated control plants (TC) 6.80, ICAR-FUSICONT treated plants (TFTR) 5.80 and *Foc* TR4 treated control (TF) plants 4.20.

During one month of planting in the control treated with *Foc*TR4 plants, the disease progressed and the early symptoms of older leaf yellowing appeared in the infected plants. With time, unimmunized plants inoculated with *Foc* TR4 (TF) exhibited mortality between 60 to 90 days. At 90th day after planting, the treatment TF showed a significant (*p* ≤ 0.05) high mean disease severity index (DSI) of 3.45 whereas, the bio-immunized plants (TFBI) and plants treated with ICAR-FUSICONT (TFTR) showed significant (*p* ≤ 0.05) lower DSI (0.20 and 1.00) compared to *Foc* TR4 treated control (TF) (Table 2).

### 3.4 Analysis of defense-related enzymes and phenol content

**Figure 3:**
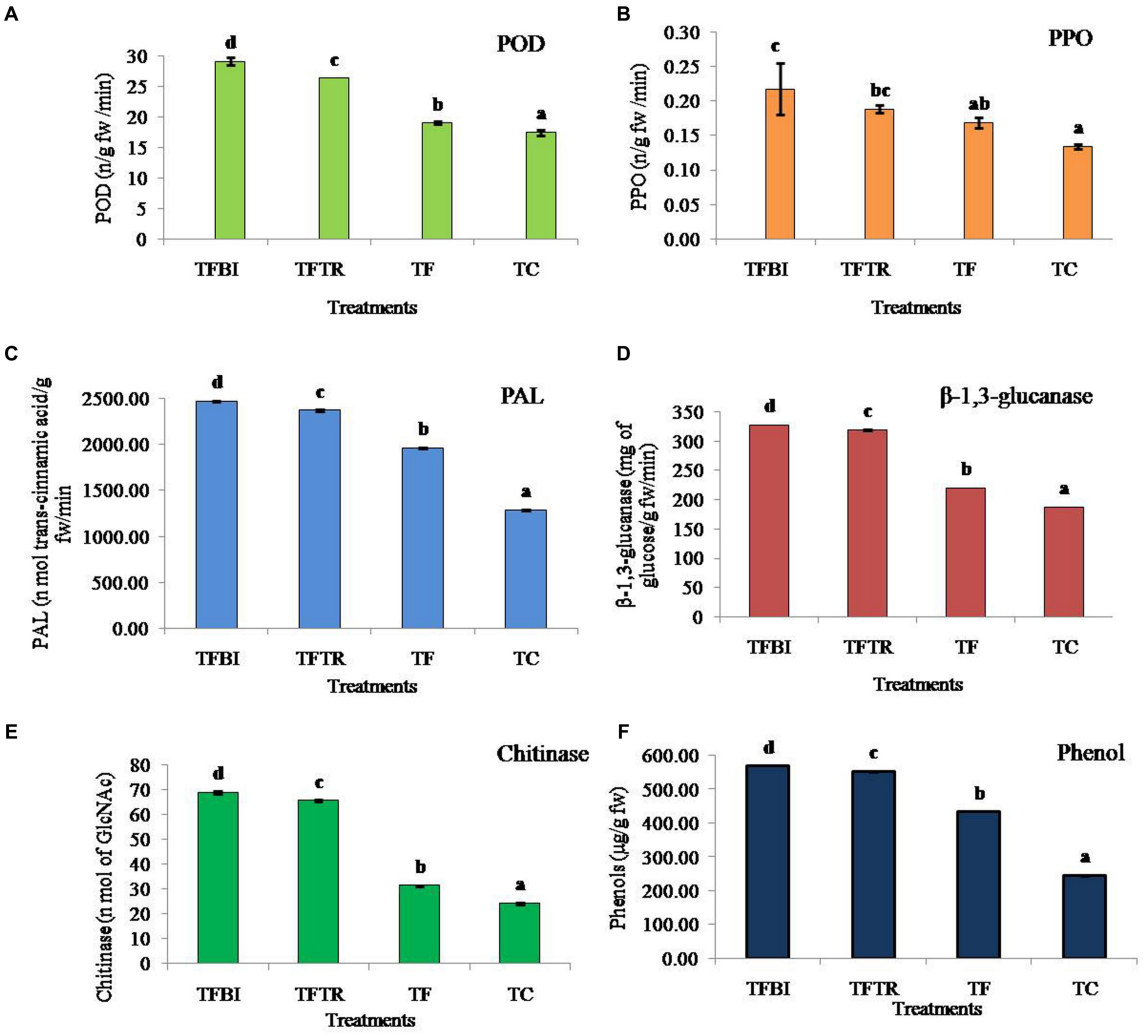
Effect of treatments of on different antioxidant and defense enzymes **(A)** Peroxidase (POD) (ng^−1^ fresh wt.), **(B)** Polyphenol oxidase (PPO) (ng^−1^ fresh wt.), **(C)** Phenylalanine ammonia lyase (PAL) (nmol of trans-cinnamic acid g^−1^ fresh wt. min^−1^), **(D)** β-1,3-glucanase (mg of glucose g^−1^ fresh wt. min^−1^), **(E)** Chitinase (nmol of GlcNAc g^−1^ fresh wt. min^−1^), and **(F)** Phenol (μg g^−1^ fresh wt.) of banana plants of G9 variety. Where, T1 = bio-immunized plants with *Foc* TR4 (TFBI), T2 = unimmunized plants with *Foc* TR4 treated with *Trichoderma* (TFTR), T3 = unimmunized plants with *Foc* TR4 (TF), and T4 = unimmunized plants without *Foc* TR4. Values are mean of three replicates with ± standard deviation. Means denoted by different letters indicate significant difference between the treatments (*p* ≤ 0.05) (DMRT).

Comparing bio-immunized plants to unimmunized plants, a significant increase in the production of all the analyzed enzymes, including phenylalanine ammonia lyase (PAL), β-1,3- glucanase, polyphenol oxidase (PPO), peroxidase (POD), phenol, and chitinase was observed (Figure 3). However, compared to the *Foc* infected hybrids, the control (uninoculated) plants usually had reduced enzyme activity. The enhanced antioxidant enzyme activities and PR proteins in Foc inoculated plants showed that the *Foc* concentration led to stress, which was detrimental to plant cells and increased antioxidant and reactive active scavenging systems indicating activation of host’s defense mechanism.

The POD enzyme activity in banana leaves was significantly different among the four treatments (*F* = 599.26, df = 3, 8, *p* = 0.00). Both the treatments TFBI and TFTR led to a significant activity increase by 34.76 and 27.91% when compared with the *Foc* TR4 treated control (TF) similarly, increased by 39.98 and 33.67% compared to untreated control (TC) respectively. The activity of PPO varied significantly (*F* = 10.27, df = 3,8, *p* = 0.004) by 22.73% (TFBI), 10.53% (TFTR) compared to *Foc* TR4 inoculated unimmunized plants (TF), and by 40.91% (TFBI), 31.58% (TFTR), and 23.53% (TF) compared to *Foc* TR4 uninoculated control plants (TC). The PAL activity ranged from 1963.00 to 2472.25 nmol. Of trans-cinnamic acid g-1 fw min-1 in *Foc* TR4 treated plants (TFBI – TF). Whereas, 1286.05 nmol. Of trans-cinnamic acid g-1 fw min-1 was observed in *Foc* TR4 uninoculated control (TC) plants. Significantly higher (*F* = 34787.98, df = 3,8, *p* = 0.00) PAL activity was observed in bio-immunized*Foc*TR4 inoculated plants (TFBI) 2472.247 nmol. Of transcinnamic acid g-1 fw min-1 followed by ICAR-FUSICONT treated plants (TFTR) 2373.363 nmol. Of transcinnamic acid g-1 fw min-1, and *Foc*TR4 inoculated control plants (TF) 1963.003 nmol. Of transcinnamic acid g-1 fw min-1, respectively. The β-1,3-glucanase activates in banana leaves of the TFBI, TFTR and TF treatments were significantly (*F* = 115509.2, df = 3,8, *p* = 0.00) increased as compared with the control (TC) (Figure 3). However, the highest β-1,3-glucanase activity was recorded in TFBI, showing 32.92% increase followed by TFTR (31.04%) relative to the *Foc*TR4 inoculated control (TF). The least β-1,3-glucanase activity 187.66 mg of glucose g-1 fw min-1was observed in *Foc*TR4 uninoculated control (TC) plants. The same pattern of antioxidants was seen for chitinase enzyme and phenol production. The chitinase activity varied significantly (F = 115509.2, df = 3,8, *p* = 0.00) by 54.42% (T 1), 49.60% (TFTR) compared to *Foc*TR4 inoculated control (TF), and by 64.70% (TFBI), 62.92% (TFTR), and 22.57% (TF) relative to the *Foc*TR4 uninoculated control (TC) plants. The phenol quantity varied significantly (*F* = 172980.7, df = 3,8, *p* = 0.00) by 23.89% (TFBI), 21.16% (TFTR) compared to inoculated control (TF).

### 3.5 Scanning electron microscopy analysis of different treatments *FOC* TR4 challenge inoculation and bio-immunization

**Figure 4:**
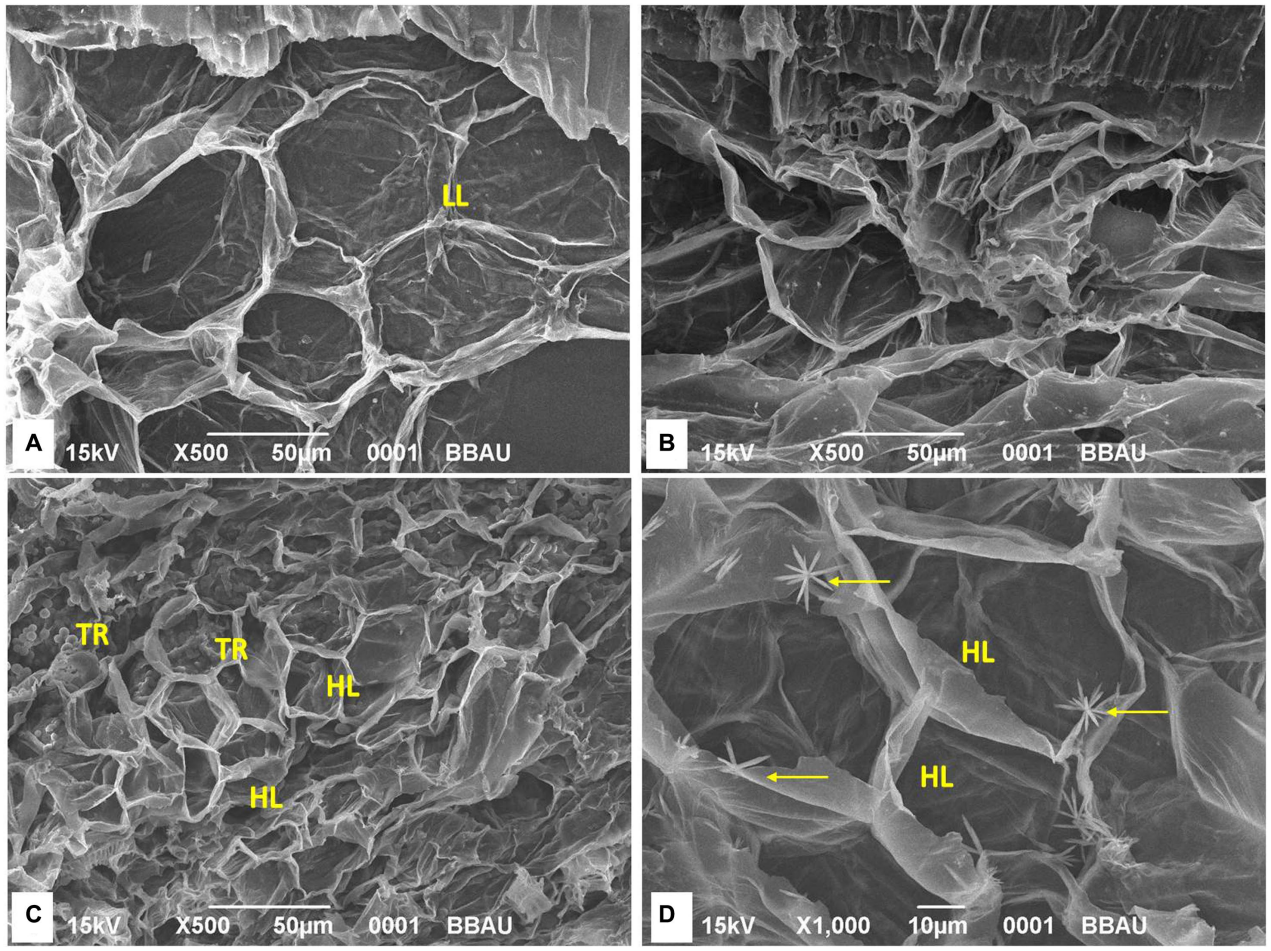
Scanning electron microscopy (SEM) analysis of different treatments. Lignification of the cells has been identified in the treatment TFTR and TFBI while tissues were found to be damaged in TF and lesser lignifications of cells has been noticed in TC. SEM images indicates that lignifications of cells has been induced both in TFTR and TFBI speculating that the metabolite induced lignin biosynthesis could be operating in conferring tolerance against the pathogen Foc invasion. **(A)** TC, Treatment Control; **(B)** TF, Treatment *Fusarium* alone; **(C)** TFTR, Treatment Fusarium+*Trichoderma reesei*; **(D)** TFBI, Treatment Fusarium+Bio-Immunizer. Where markings in yellow indicate intensity of lignifications. LL, Low lignifications; HL, High lignifications; TR, *Trichoderma reesei*.

SEM analysis of four treatments indicated that the lignifications of the cells have been identified in the treatment TFTR and TFBI. Quite contrarily, the tissues were found to be damaged in TF, and lesser lignified cell membranes were observed in the control TC. The close-up view of the cells in TFTR and TFBI indicates the metabolite induced lignin biosynthesis which could be one of the adaptive mechanisms conferring tolerance against the pathogen *Foc* invasion (Figure 4).

### 3.6 Metabolic profiling of bioactive metabolites in whole plant samples of banana plantlets under different treatments of *FOC* TR4 challenge inoculation and bio-immunization by using LC-MS analysis

**Figure 5:**
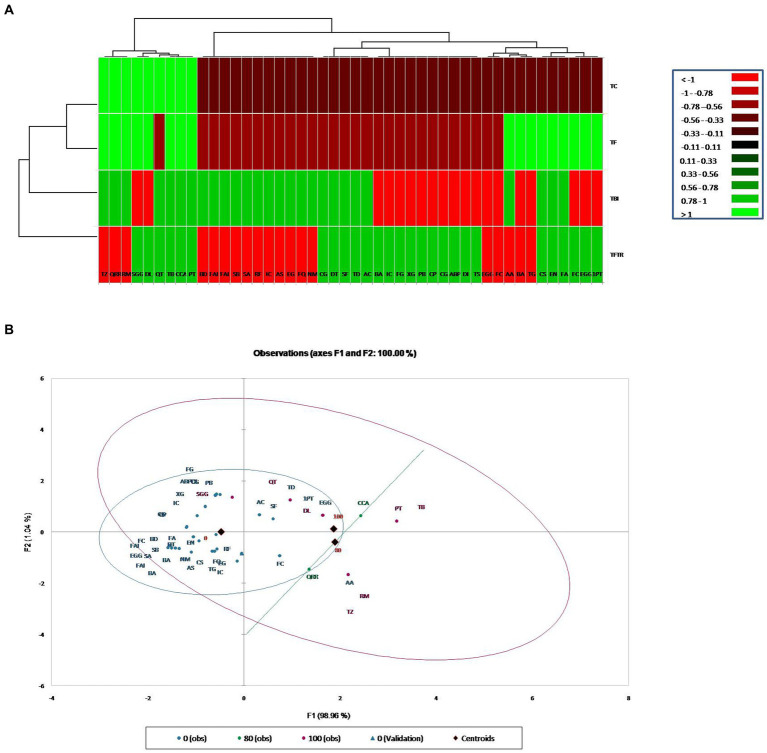
Cluster analysis for classification and categorization of metabolites obtained in the LC-MS data pooled from the four treatments. **(A)** Heat map showing profile of different metabolites expressed in banana plants under different treatments along with vertical and horizontal clustering marked on the top and left panels. *X*-Axis denotes metabolites and *Y*-axis represents treatments. **(B)** Principal component analysis showing clustering of metabolites into different groups generated through XLSTAT software. Treatments: TC, Treatment Control; TF, Treatment *Fusarium* alone; TFBI, Treatment Fusarium+Bio-Immunizer; TFTR, Treatment Fusarium+*Trichoderma reesei.* Metabolite: TD, Tomatidin; FQ, Flavonoid quercetin; NM, Neodiosmin; EG, Epigallocatechin gallate; CCA, Cryptochlorogenic acid; QRR, Quercetin-glucoside-rhamnoside-rhamnoside; AS, Acteoside; AC, Anthocyanin; IC, Isocytric acid; RM, Rhamnose; RF, Rushflavanone; PT, Phloretin; SF, Sinefungin; DT, Dehydrotomatine; TZ, Trans Zeatin; QT, Quercetin; TB, Thebaine; EN, Enniatin A; CG, Corilagin; FA, Fusaristatin A; AA, Arachidonic acid; CS, Chlamydosporal; Sa, Surfactin isomar A; SB, Surfactin isomar B; FAI, Fengycin A isomar; BD, Bacillomycin D isomar; SGG, Soyasapagenol E-3-O-rhamnosyl glucosyl glucuronide; DI, Diglucoside isomer; DL, Demethyloleuropein; TS, Tabersonine; ABP, Α-D-Glucose-1,6-bisphosphate; CG, Catechin-0-gallate; CP, β caryophyllene; PB, Peptaibols; XG, Xyloglucan; FG, Fenigycin; IC, Iturin-C19; EGG, Epi gallocatechin-O-gallate; 1PT, 1-Palmitoyl-2-10-hydroxy-5,8,11-tridecatrienoic acid; BA, Beauveric acid; FC, Fusarin C; EGG, Epi gallocatechin-O-gallate; BA, Beauveric acid; FC, Fusarin C; TG, Trigalloyl glucose.

Metabolic profiling of whole plant extracts of banana plantlets under different treatments by LCMS showed a significant increase in the synthesis of bioactive compounds classified mainly as flavonoids, quinones, phenolic acids, and derivatives of lipids and amino acids in bio-immunized treatment than the others. A total of 27 bioactive compounds with repeated peaks of high intensity were characterized in the treatment TFBI involving bio-immunization and *Foc*TR4 challenge inoculation, 28 with the treatment TFTR involving *Trichoderma reesei* isolate CSR T-3 and challenge inoculation with *Foc* TR4and 17 under treatment TF comprising of *Foc*TR4 challenge inoculated plantlets (Table 1). In the untreated control TC involving plantlets that were neither bio-immunized nor challenge inoculated with *Foc*TR4 only nine bioactive compounds comprising carbohydrate, phenol derivatives with high-intensity peaks were detected. The heatmap with linkages (Figure 5A) distinctly separates the TFBI and TFTR treatments with TF and TC with dissimilarity of 74.39%. The PCA analysis of the detected metabolites showed that the differentially accumulated metabolites (DAM) grouped distinctly with the respective treatments. The first and second principal components depicted 47.04 and 27.36% variation, respectively (Figure 5B). The divergence of the vectors of the TFTR treatment involving *Trichoderma reesei* CSR T-3 isolate and TFBI involving the bio-immunization from the PCA signifies the higher contribution of the PC (principal components) while the convergence of the vectors of TF treatment with *Foc* TR4 alone and TC signifies the least contribution of the PC (Figure 5B). We observed five differentially accumulated metabolites (SF Sinefungin, AA Arachidonic acid, QRR Quercetin glucoside rhamnoside-rhamnoside, RM Rhamnose, and TZ Trans zeatin) in treatment TBI involving bio-immunization and *Foc* TR4 challenge inoculation whereas the presence of these compounds were not observed in other treatments.

### 3.7 Multi-location trial and assessment of the performance of bio-immunized plants

Field trials were conducted for two consecutive years (2020–21 and 2021–22) in the field of susceptible soil at two locations Nirpur, Purnia district, and Dighari, Katihar district (Bihar, India) (Figure 1). It was observed that bio-immunized plants significantly (*p* ≤ 0.05) enhanced the growth and yield compared to unimmunized plants. At location 1, the maximum plant height and plant girth were recorded in TBITR (257.30 cm, 70.80 cm) followed by TBI (249.30 cm, 68.70 cm) and TTR (238.90 cm, 57.95 cm) compared to untreated control plants (TC) (222.65 cm, 53.65 cm), respectively. Similarly, each treatment caused a significant (*p* ≤ 0.05) increase in no. of leaves/plant, 3rd leaf length, and breadth compared to control as indicated by Duncan’s multiple range tests. The highest increase in no. of leaves/plant (31.39%), 3rd leaf length (20.04%), and 3rd leaf breadth (19.62%) in TBITR were significantly higher than that found in TBI (23.5, 16.07, and 17.78%), TTR (5.56, 6.86, and 9.66%) compared to control (TC). Based on DMRT analysis bunch weight, no. of hand/bunch, and no. of finger/hand were statistically (*p* ≤ 0.05) higher for the treated plants compared to control (Table 3). At location 1, the highest bunch weight (30.85 kg), and no. of hand/bunch (12.30) was observed in treatment TBITR whereas, a maximum no. of fingers/ hand was found in TBI (17.50) significantly similar to TBITR (17.45) followed by TTR and TBI, respectively.

**Figure 6:**
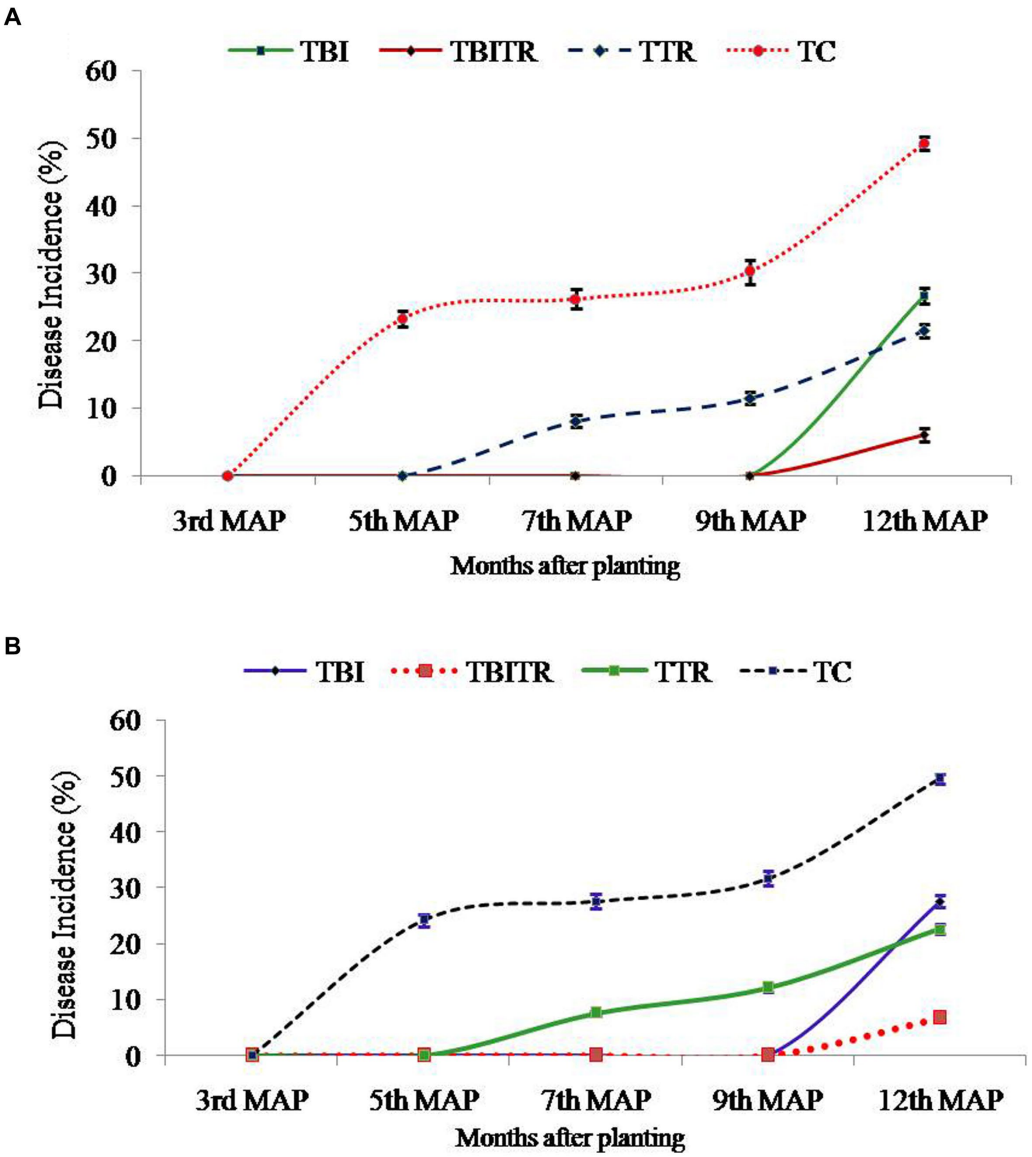
Disease incidence analysis of *Foc* TR4 in Grand Naine (G9) banana plants throughout the two year field trial at two different locations **(A)** Nirpur, Purnia district, Bihar, India and **(B)** Dighari, Katihar district, Bihar, India under infected fields.

At location 2, the highest plant height was observed in TBITR (249.40 cm) and plant girth in TBI (66.45 cm), which were significantly (*p* ≤ 0.05) higher than control (TC). The maximum no. of leaves/plant, 3rd leaf length (12.35 and 228.35 cm) was found in TBITR, while the highest 3rd leaf breath (82.60 cm) was recorded in TBI significantly (*p* ≤ 0.05) higher than other treatments and control as indicated by DMRT. Similarly, the highest bunch weight (31.70 kg), no. of hand/bunch (12.40), was observed in TBITR inoculated plants, while the maximum no. of finger/hand (18.95) was observed in TBI followed by TBITR, TTR, and control (TC), respectively. Percentage disease incidence was calculated for all the treatments planted at two locations in Nirpur, Purnia district, and Dighari, Katihar district (Bihar, India) over the years 2020–21 and 2021–22 (Figure 6). At location 1, in the case of control (TC) the appearance of the disease started after 3rd month of planting, at the final growth stage (12th MAP) the mean incidence recorded was 49.50%. In the case of CSR-T-3 (TTR) treated plants the incidence was found (21.50%) on the 12th MAP and the appearance of the disease started after the 5th month after planting. However, in the case of bio-immunized plants (TBI) disease appearance started after the 9th month after planting with a disease incidence was 26.70% whereas, when we combine bio-immunized plants along with rotational application of CSR-T-3 (TBITR) appearance of disease started at 9th month after planting like TBI but, disease incidence lower down at (6.10%). A similar kind of trend was observed at location 2. The highest disease incidence (49.50%) was observed in control (TC) after the 12th MAP followed by bio-immunized plants (TBI) (27.60%), CSR-T-3 treated (TTR) (22.60%), and bio-immunized plants with application of CSR-T-3 (TBITR) (6.70%) respectively. In the case of TC, the disease incidence started at the 3rd MAP while, in TTR the disease incidence started at the 5th MAP. In the case of TBI and TBITR, the disease incidence started at the 9th MAP.

## 4 Discussion

Banana is one of the major important commercial fruit crops of India, known for being cultivated in Maharashtra, Karnataka, Tamil Nadu, Andhra Pradesh, Odisha, Gujarat, and parts of the North East. Recently, banana cultivation has been expanding significantly in Uttar Pradesh and Bihar covering about 69,380 ha and 31,070 ha, respectively (Damodaran et al., 2021). In the recent past, the cultivation of commercial cultivar G9 of banana has been threatened by the outbreak of a virulent strain of *Fusarium* wilt tropical race 4. The pathogen is known for its rapid proliferation and devastation of the plantation within a short period causing a huge economic loss to growers. Control of the disease has been a great challenge to researchers across the globe to date. In an attempt toward successful management, the complex polycyclic nature of the pathogen and its ability to mutate into different Vertical Compatibility Groups (VCG’s) warranted the need for supportive technologies to build resistance in the host. According to Chow et al. (2008) engineered peptide-based biopolymers have recently attracted much attention as a new class of materials. One such effort resulted in the development of a lipo-polypeptide-based biomolecule from *Trichoderma reesei* and *in vitro* bio-immunization technology. To the best of our knowledge, no single report is presently describing lipo-polypeptide-based biomolecule used in the process of bio-engineering into the banana plantlets (bio-immunization) in tissue culture root regeneration media. *Trichoderma* spp. is known as a potential biocontrol agent (El Komy et al., 2015). The metabolites secreted by *Trichoderma* spp. suppresses the plant pathogenic microorganisms and enhances plant growth (Kubicek et al., 2001; Contreras-Cornejo et al., 2015). *Trichoderma* spp., considered a potent biocontrol agent, that can survive under stressed conditions, antagonistic against phyto-pathogenic microorganisms, induces a defense mechanism in plants, and helps in plant growth promotion by secreting several secondary metabolites (Adnani et al., 2017; Poveda et al., 2020), PR proteins (Yadav et al., 2021), enzymes (Tariq et al., 2020), siderophores, phytohormones and phosphate solubilizing enzymes (Doni et al., 2014). In our findings, we observed that bio-immune formulation significantly inhibited the growth of the pathogen *Foc*TR4 under *in vitro* conditions.

We observed that bio-immunization enhanced the *in vitro* rooting and plant growth of banana plantlets. *In vitro* bio-immunization of the host plant is an advanced technology in tissue culture to develop disease-tolerant, disease-free planting material through bio-fortification of plantlets with bio-immunizing formulation. According to Chatenet et al. (2001) tissue culture allows the production and propagation of disease-free genetically homogeneous plant material. The process can also be used to develop plants resistant to various types of stresses (Bouquet and Torregrosa, 2003). According to Pati et al. (2013), the maximum mortality of micro-propagated plants occurs during the acclimatization process as plantlets undergo several morphological, physiological, and biochemical changes. In our study, a 97.57% survival rate was observed at the primary hardening stage and 93.23% at the secondary hardening stage. Uzaribara et al. (2015) recorded a 95% survival rate during the primary hardening of red banana (*Musa acuminate*) whereas, Rai et al. (2014) reported a 96% survival rate. However, mortality of plantlets may be due to injuries to the root system during transferring and sudden exposure to the harsh environment. Successful acclimatization and survival rate in banana plantlets (80–100%) under *in vitro* greenhouse conditions has been reported by several scientists (Rahman et al., 2013; Ahmed et al., 2014; Hossain et al., 2016). In the current study, the technology of *in-vitro* bio-immuniztion for producing disease-free, *Fusarium* wilt-tolerant banana plantlets were tested for its bioefficacy through a pot culture experiment where the bio-immune treated plantlets exhibited significantly low disease index (0.67) compared with the *Foc* TR-4 challenge inoculated control that showed 3.78 disease index signifying the host induced tolerance due to bio-immunization. It also significantly enhanced the plant growth compared to the control due to the presence of PGPR properties of *Trichoderma reesei* in the bio-immunized plantlets. According to Cai et al. (2015) *Trichoderma* spp. have been reported to increase plant growth by releasing several hormones that enhance root development and plant growth, which in turn increases the secretion of root exudates resulting in the availability of nutrients for microbial growth (Elad, 2000; Carvalhais et al., 2015). In the same line, Yedidia et al. (2001) reported that *Trichoderma* spp. increases the length of primary and lateral roots which results in enhanced nutrient uptake by the plants. They exert biocontrol action against fungal phytopathogens by inducing resistance in host plants and promoting plant growth (Fiorentino et al., 2018; Zhao et al., 2020). *Trichoderma* sp. was found to enhance the concentration of essential nutrients in the shoots and roots of cucumber and tomato seedlings (Azarmi et al., 2011).

The results obtained in this study revealed that *in vitro* bio-immunization of banana plantlets during organogenesis through the bio-immune formulation of *T. reesei* showed better activity that increased defense-related enzymes PAL, PPO, POD, β-1,3-glucanase, chitinase, and phenolics by a greater amount. Our findings are in line with Damodaran et al. (2020), who reported that *T. reesei* enhanced β-1,3-glucanase, POD, chitinase, PPO, and PAL with higher phenolic contents. Singh et al. (2021) reported that *Trichoderma* spp. showed significantly higher defense-related enzymes such as PAL, POD, and PPO. Joseph et al. (1998) reported that POD inhibited the mycelia growth and spore germination of *Pseudocercospora abelmoschi* and *P. cruenta.* Kavino et al. (2007b) showed enhanced POD and PAL activity in bacterial strain-treated banana plantlets against *Banana bunchy top virus.* Another enzyme PPO is known to catalyze the oxidation of phenolic substances into highly reactive quinines (Araji et al., 2014). Karthikeyan et al. (2006) reported that a consortium of *Trichoderma viride* with *Pseudomonas fluorescens* enhanced the PPO activity in coconut plants against *Ganoderma*. Consortia of *Trichoderma* spp. and with other bacterial isolates have been reported to increase the defense enzyme activity of PAL, POD, and PPO during pathogen invasion (Jain et al., 2012; Singh et al., 2013; Singh and Singh, 2014). Peroxidase (POD) is one of the most important enzymes that is responsible for lignin synthesis and developing plant resistance against phytopathogen (Bruce and West, 1989) and restricts fungal growth (Macko et al., 1968). Several scientists reported that POD activity was elicited during the pathogen infection in cucumber (Yedidia et al., 1999; Chen et al., 2000), tomato (Nandakumar et al., 2001; Ramamoorthy et al., 2002), banana (Damodaran et al., 2020), and brinjal (Singh et al., 2021). Similarly, a significantly higher amount of PAL activity was observed in brinjal (Singh et al., 2021), chickpea (Singh et al., 2013), pea (Jain et al., 2012), tomato (Singh and Singh, 2014) and banana (Damodaran et al., 2020). Phenolic compounds present in plants have antimicrobial properties and serve as signaling molecules (Hammerschmidt, 2005).

**Figure 7:**
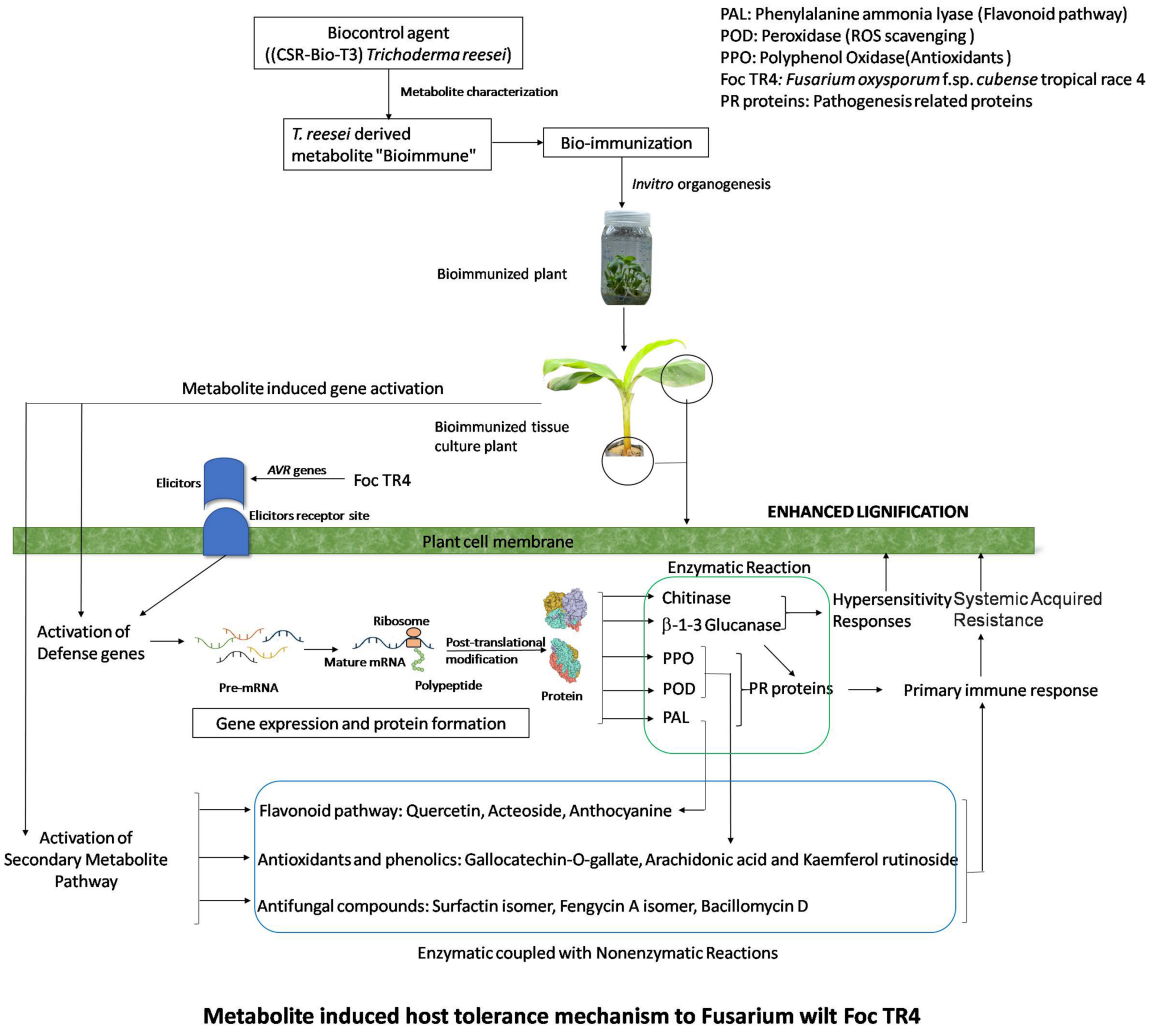
Metabolite induced host tolerance mechanism to Fusarium wilt *Foc* TR4. Bioimmunized plants showed improved host defense mechanism (HDM). HDM is activated by enhanced secondary metabolite pathway, ROS scavenging enzymes, PR proteins which lead to lignifications of cells.

Our findings from secondary metabolite profiling using LCMS have furthered the host tolerance attributed by bio-immunization technology by confirming the role of antifungal secondary metabolites present in the *Trichoderma reesei* isolates CSR-T-3contributing to the suppression of *Foc*. The versatile nature of antifungal secondary metabolites from endophytic *Brachybacterium paraconglomeratum* isolated from the resistant cultivar YKM5 was proved to be responsible for the suppression of *Foc* under *in vitro* conditions (Ravi et al., 2022). In the current study, we observed high expression of antioxidants like Quercetin and Quercetin rhamnosyl-rutinoside to the level of intensity 100 and 80%, respectively, in the bio-immunized TBI treatment challenge inoculated with *FOC* TR4. Likewise, co-culturing of *B. velezensis* YRBBR6 along with *Foc* KP (Pisang Awak strain) induced the secretion of secondary metabolites, *viz.*, dihydroacridine, nonanol from the antagonistic bacteria inhibit plant pathogens and promote plant growth (Khalid and Keller, 2021). Quercetin isoflavonoids known to have antibacterial and antiviral activity are induced in plant systems under stress conditions to enable immune response (Johari et al., 2012; Lachowicz et al., 2020). The challenge inoculation of *Foc* TR4 significantly shows the presence of fungal toxins of *Fusarium* sp. like Fusaristatin A, Fusarin C, and Beauveric acid signifying the intense activity of pathogen in the plant system due to *Foc* TR4. However, the presence of fungal toxins was found to be at a lower level running from (4 to 22%) intently signifying the role of bio-immunization in TBI treatment in the suppression of toxins produced by *Foc*TR-4 in the presence of the antifungal metabolites. Besides this, the intensity of lignifications in the cell walls of bio-immune (TFBI) and *Trichoderma reesei* treatment (TFTR) indicates the mechanism of induction of host defense through signal transduction and regulation of lignin biosynthesis pathway. The mechanism of host defense conferred by the bio-immune molecule is explained in Figure 7 wherein it is speculated that the bio-immunized plants exhibited activation of the host defense system in terms of enhanced secondary metabolite production pathway, pathogenesis-related proteins (PR proteins) and reactive oxygen species scavenging system (ROS scavengers) by the production of antifungal compounds, antioxidants, and phenols, besides enhanced lignification in the cells (evident from SEM images, Figure 4).

Field performance of the different treatments of banana plants also indicated that TFBI outperformed the other treatments in terms of yield, bunch weight, and plant growth characters. The expression of disease symptoms is also very negligible in these two treatments thus proving that this strategy is a rationalized field translational success report of *in vitro* Bio-immunized plants for the management of Fusarium wilt disease of banana. Earlier, Damodaran (2018) demonstrated successful control of *Foc* TR4 using ICAR-FUSICONT during the late stages of plant growth whose principal microorganism is *Trichoderma reesei*. This report is an advancement over the previous report, in which earlier days of plant growth as well as infections during the initial 4-5 months are also taken care of; as ICAR-FUSICONT works well in the later stages.

## 5 Conclusion

Over the past decade, Fusarium disease has become a serious problem for banana growers not only in terms of economic loss but also because of the disposal of diseased plants. The major setbacks that affect the management of the disease include a lack of information on the resistant gene pools, no effective chemical control, and a lack of virulent biological control agents. However, in our previous study, we had identified *Trichoderma reesei* as a potential biological agent for the management of the pathogen strain. We report here another novel approach of metabolite-induced host immune system for combating the Fusarium wilt disease of banana. This study is a holistic approach to a technological intervention that has been validated in the field for the management of Fusarium disease in banana (*Foc* TR4).

## Data availability statement

The original contributions presented in the study are included in the article/Supplementary material, further inquiries can be directed to the corresponding authors.

## Author contributions

TD conceived, planned, and designed the experiments. MMi conducted the *in vitro* lab experiments and contributed to manuscript preparation with TD. MMu designed, analyzed the LC-MS, enzyme analysis, field data, and interpreted the results for elucidation of mechanism. SR designed the field experiments, data collection, performed the statistical analysis of the data. KY, SKi, and AK did *in vitro* Bioimmunization work. PD, PB, and RG performed the experiments of dual culture, pot, and field experiments. SK performed LC-MS, enzyme analysis, data interpretation, and prepared conceptual mechanism figure. All authors contributed to the article and approved the submitted version.
